# Fragmentation and bond strength of airborne diesel soot agglomerates

**DOI:** 10.1186/1743-8977-5-9

**Published:** 2008-06-04

**Authors:** Sonja Rothenbacher, Armin Messerer, Gerhard Kasper

**Affiliations:** 1 Institut für Mechanische Verfahrenstechnik und Mechanik, Universität Karlsruhe (TH), 76128 Karlsruhe, Germany; 2 Institute of Hydrochemistry, TU München, 81377 Munich, Germany; 3 Mahle GmbH, 70376 Stuttgart, Germany

## Abstract

**Background:**

The potential of diesel soot aerosol particles to break up into smaller units under mechanical stress was investigated by a direct impaction technique which measures the degree of fragmentation of individual agglomerates vs. impact energy. Diesel aerosol was generated by an idling diesel engine used for passenger vehicles. Both the aerosol emitted directly and aerosol that had undergone additional growth by Brownian coagulation ("aging") was investigated. Optionally a thermo-desoption technique at 280°C was used to remove all high-volatility and the majority of low-volatility HC adsorbates from the aerosol before aging.

**Results:**

It was found that the primary soot agglomerates emitted directly from the engine could not be fragmented at all. Soot agglomerates permitted to grow additionally by Brownian coagulation of the primary emitted particles could be fragmented to a maximum of 75% and 60% respectively, depending on whether adsorbates were removed from their surface prior to aging or not. At most, these aged agglomerates could be broken down to roughly the size of the agglomerates from the primary emission. The energy required for a 50% fragmentation probability of all bonds within an agglomerate was reduced by roughly a factor of 2 when aging "dry" agglomerates. Average bond energies derived from the data were 0.52*10^-16 ^and 1.2*10^-16 ^J, respectively. This is about 2 orders of magnitude higher than estimates for pure van-der-Waals agglomerates, but agrees quite well with other observations.

**Conclusion:**

Although direct conclusions regarding the behavior of inhaled diesel aerosol in contact with body fluids cannot be drawn from such measurements, the results imply that highly agglomerated soot aerosol particles are unlikely to break up into units smaller than roughly the size distribution emitted as tail pipe soot.

## Introduction

Diesel soot aerosol particles are usually agglomerated to varying degrees, depending on whether they have freshly emerged from the tailpipe, or whether they are aged (i.e. have undergone collisional growth) as in the case of prolonged residence times in a street tunnel. The mechanical stability of soot agglomerate particles is of considerable interest. When used as dye, easy disintegration of "lamp-black" in liquid suspension is usually a desired property; as a reinforcement in automobile tires it is generally not. Shear flow induced soot reentrainment in diesel particulate filters has a strong influence on the over-all system performance [1]. In conjunction with the assessment of health risks posed by inhaled diesel soot in contact with tissue and body fluids, the potential for breaking up larger agglomerate structures into smaller units may have various implications, as well.

Soot, as well as many other anthropogenic or "engineered" nanoparticulate aerosols, is formed in a series of chemical and physical growth processes involving nucleation from supersaturated vapor and coagulation. These initial steps can be accompanied by secondary structural transformations such as condensation of unburned fuel and/or lubricant oil vapors onto the freshly formed, fractal agglomerates [2], some of which may actually be removed again during thermal after-treatment of the soot, for example by oxidation catalysts. After its release into the atmosphere, the diesel aerosol will generally undergo further changes. For example it may undergo collision growth by Brownian coagulation due to slow dilution in confined spaces such as tunnels.

Each of these processes has implications on the stability of a soot agglomerate, and hence on the likelihood of its breaking up later on, under mechanical stress or electrochemical actions in fluid suspension. While pure Brownian coagulation of dry particles will result mostly in van der Waals bonding, the various secondary processes are expected to cause a significant increase in adhesion strength due to the formation of solid or liquid bridges. Hence a soot agglomerate with a complex history may actually consist of substructures with different degrees of mechanical stability. Almost no information is available however on how the various stages of soot formation might affect the strength of individual diesel soot particles. The vast majority of studies concern downstream issues such the effects of carbon black fillers on the tensile strength of rubber. Indeed Friedlander [3] suggested links between agglomerate structure and polymer behavior, and some measurements in this direction have been made lately with AFM techniques [4].

Recently [5,6] a method was described for measuring the probability of fragmentation of agglomerated aerosol nanoparticles by direct impact from an aerosol stream onto a solid substrate (usually a TEM grid). The method is based on a modified single-stage low pressure impactor (SS-LPI). Particles impact at variable but defined velocities and the degree of fragmentation is determined by image analysis of individual particles in the deposit. The paper also presented first data on the fragmentation probability of nano-agglomerates of silver, nickel, titanium as a function of impact energy. While it does not give a direct measure of interparticle forces, the technique has proven useful to quantify changes and relative magnitudes of effects. For example, one can show the influence of bond strength manipulations on polystyrene particle adhesion [7], or of gradual sintering on the degree of fragmentation of silica nano-aerosols [Seipenbusch, M, Rothenbacher S, Kirchhoff M, Schmid HJ, Weber AP and Kasper G: Interparticle forces in silica nanoparticle agglomerates (submitted)]. These results will be discussed later, in the context of experimental results. We also know that thermally induced rearrangement of primary particles within an agglomerate requires less energy than break-up [8]; experiments have furthermore shown that this rearrangement can be slowed by modification of the nanoagglomerate surface [9]. Generally however, quantitative data on the strength of agglomerate aerosol particles (other than soot) are either scarce or inexistent, especially in the nano size range.

In this paper, we present exploratory results obtained with the above mentioned technique on the fragmentability of soot aerosol particles produced by a passenger vehicle diesel engine, and on the effect of an exhaust after-treatment on the agglomerate strength. The after-treatment was simulated by a thermal denuder, which removes the majority of the adsorbed hydrocarbons at a temperature of 280°C [10]. Since it became clear very quickly during the experiments, that the primary diesel aerosol from the engine exhaust cannot be fragmented at all – with or without removal of adsorbates – the initial question was modified to investigate the effect of adsorbates on an aged diesel aerosol. The aging was done by giving the primary aerosol time to coagulate.

### Experimental Setup, Techniques and Aerosol Characteristics

#### Exp. set-up and aerosol

The diesel soot aerosols were taken from the exhaust gas of an idling Volkswagen 1.4 l TDI (55 kW) engine operated with diesel fuel of a sulfur content below 10 ppm. We know from a detailed analysis of soot and exhaust gas composition for a comparable Volkswagen TDI engine operated under comparable idling conditions that the organic carbon (OC) mass fraction of the soot ranged between 10 and 25% [11].

**Figure 1:**
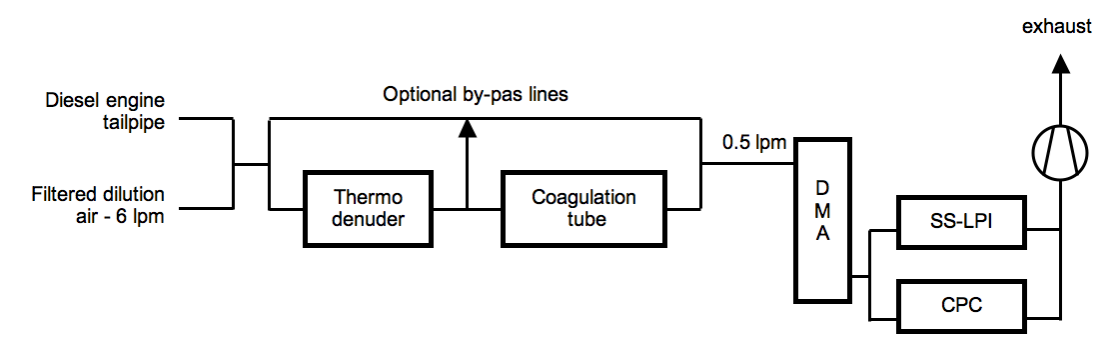
***Experimental setup ***for generation, thermal after-treatment and agglomeration of diesel soot (with optional by-pass lines). The devices to the right are used for the subsequent measurement of agglomerate strength. (DMA = differential mobility analyzer, SS-LPI = single stage low pressure impactor; CPC = condensation particle counter) The DMA operates at a fixed setting; the CPC is used to monitor the concentration only.

Fig. 1 shows a schematic diagram of the set-up used for the fragmentation experiments. The engine exhaust gas was diluted 12:1 with dry air to avoid condensation of water in the sampling lines. Under idling conditions the condensation of the volatile organic fraction onto the soot particles occurs in the exhaust system and is therefore not significantly influenced by the dilution. For the experiments with thermal after-treatment, the aerosol was led into the thermodenuder (TD) developed at the Institute of Hydrochemistry, TU Munich. In the TD the diluted aerosol could be heated to 280°C and then drawn into a desorber tube consisting of a perforated stainless steel inner tube surrounded by a filling of activated charcoal grains. The tube geometry (i.d. 10 mm, length 0.6 m) gave an aerosol residence time of 0.43 s. In thermodenuders the highly volatile organic carbon (OC) fraction attributed mainly to fuel hydrocarbons and their fragments is removed between 30 and 175°C, low volatility OC mainly stemming from lube oil emissions is removed between 175 and 300°C [12]. Under the operating conditions described above, the TD removes the highly volatile and the majority of the low volatility OC whose fraction is determined by the complex interaction of engine operating conditions and lube oil properties (Dronia, 2003) [10]. For the experiments without thermal treatment, a bypass line with comparable aerosol residence time was used. Downstream of the thermodenuder, the diesel soot aerosol could be aged by Brownian coagulation in a flow tube (not shown in Fig. 1) with a residence time of 12 s, which suffices to produce a modest degree of additional agglomeration.

**Figure 2:**
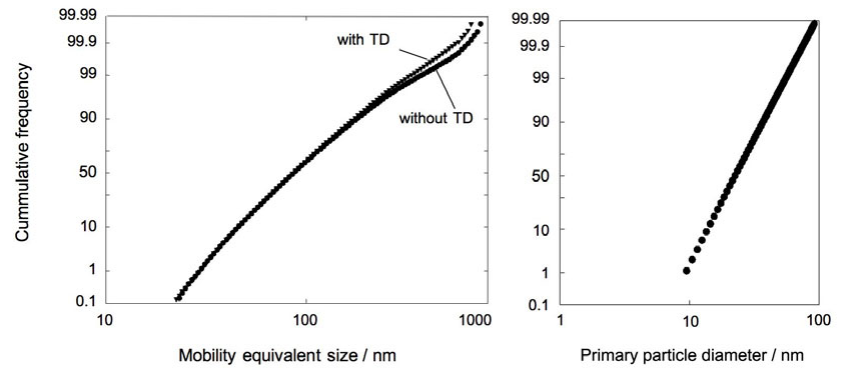
***Cumulative number size distributions ***of agglomerates (left) and primary particles (right) of the diesel aerosol measured by electrical mobility and TEM, respectively. Distributions with and without the thermodenuder are almost indistinguishable.

Fig. 2 (left) shows a cumulative mobility size distribution of the agglomerated diesel soot particles, with and without TD. The total number concentration of the aerosol bypassing the denuder was about 10^5^cm^-3^. The agglomerates passing the denuder showed a mean mobility diameter of 94 nm and geom. standard deviation of 1.7, the ones without denuder treatment of 96 nm, indicating that the TD did not affect the size distribution noticeably, despite losses on the order of 30% in the denuder.

Fig. 2 (right) shows a representative size distribution of the primary particles without thermodenuder, obtained by image analysis of 2000 primary particles from transmission electron micrographs. The count mean particle size is 22 nm with a standard deviation of 1.48, which is in good agreement with earlier investigations [13,14]. No significant difference in primary size distribution was detected between the treated and untreated particles, emphasizing again that the primary particle generation process is terminated at the engine outlet.

**Figure 3:**
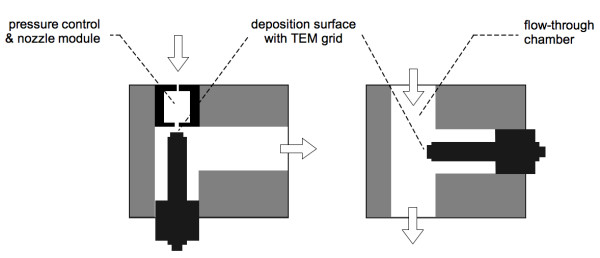
**Schematic diagram of fragmentation apparatus** – LHS: operated as single-stage low pressure impactor with nozzle module inserted and impaction surface (TEM grid holder) facing the accelerating nozzle; RHS: operated as ambient pressure diffusional sampler with nozzle module removed and collector surface position switched.

The ***technique for fragmenting airborne agglomerates ***and determining their degree of fragmentation as a function of impact velocity has been described thoroughly in earlier papers [5,6], so that we can confine ourselves here to summarizing its essential aspects. Initially, a narrow size fraction of the diesel aerosol having a mean mobility diameter of 150 nm is selected by DMA (TSI Model 3071; operated at a flow ratio of 1:10). This fraction is then introduced to the fragmentation device, a modified single-stage low pressure impactor (SS-LPI) of the type described first by de la Mora [15]. A schematic drawing of the device is shown in Fig. 3. The impactor is generally operated at Stokes numbers well above the range typically used for size classification. Hence all particles are collected directly on TEM grids; bounce is insignificant. By varying the nozzle flow velocity via the pressure in the impaction chamber from about 30 to 300 m/s, one can adjust the impact velocity (ca. 85% of mean nozzle exit velocity [16]) and hence the degree of fragmentation.

**Figure 4:**
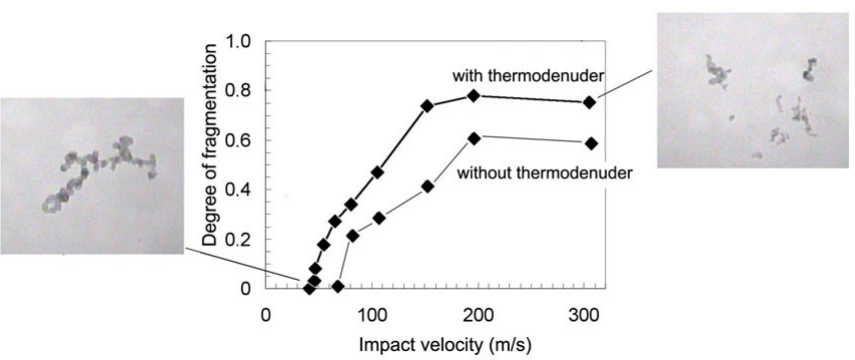
***Degree of fragmentation as a function of impact velocity ***for diesel soot particles from aged engine exhaust with and without thermal after-treatment. Inserted TEM micrographs illustrate the unfragmented state and the highest degree of fragmentation of a typical agglomerate.

The degree of fragmentation is determined by image analysis of a representative number of individual agglomerates collected on TEM grids (see Fig. 4 for illustration). Since the observed fragments have to be assigned to the original agglomerates, it is quite important to ensure appropriately low deposit densities. The inlet concentration of the SS-LPI is therefore monitored by a parallel CPC to adjust the optimal sampling time, based on experience. The degree of fragmentation, F, which we use here as a measure for strength, is defined as the fraction of broken bonds in an agglomerate. It is irrelevant whether any of the fragments are deformed, i.e. the primary particles are rearranged upon impact, as long as the components are not dislocated. We do assume however that the number of bonds can still be determined with reasonable accuracy. The degree of fragmentation is based on the total number of bonds per agglomerate particle, which must be determined without impaction. This is done by depositing agglomerates by diffusion onto TEM grids. For this purpose the impactor is operated with a different flow path with the nozzle module removed (see Fig. 4).

A point to be addressed is sampling volatile or semi-volatile materials with a low-pressure impactor. In this context Berner (private communication) has pointed out that the passage times from the entrance nozzle to the impaction surface are so short (microseconds), that no significant evaporation takes place before impact, even for water droplets.

Systematic and statistical errors in the impact velocity are on the order of a few percent at most, since the device is quite accurately controlled. A given value for the fragmentation degree is typically based on the evaluation of several hundred particle images; errors are thus estimated to be on the order of about 10%.

## Results and discussion

The first, and rather significant result of the fragmentation experiments was that soot agglomerates emitted directly from the exhaust did not break-up at all, with the exception of a few very minor fragments, even at the highest impact velocities achievable in the measurement device. This outcome was not changed by the use of a thermodenuder. It is known from the literature [17,18] that the full structural fragility of agglomerates is never recovered, even if 100% of liquid adsorbates are removed. A small residual fraction of unbreakable agglomerates has also been observed among nebulized polystyrene particles [7]. Nevertheless, it was surprising to find no effect at all.

For this reason, complete fragment analyses were only performed on agglomerates "aged" by further Brownian coagulation of the aerosol after the engine exhaust. This aging was done with and without removing some of the adsorbed hydrocarbons in the thermodenuder, in order to determine the effect of adsorbates on the cohesive strength of secondary agglomerates. The fragmentation data for the "aged" particles are shown in Fig. 4 as a function of impact velocity. According to this figure, thermally treated soot agglomerates showed a 50% fragmentation at an impact velocity 111 m/s, untreated agglomerates (not shown) at 171 m/s. For both species the maximum attainable degree of fragmentation was reached around 200 m/s with about 60% and 75%, respectively.

Clearly, the reduction of adsorbates on the particle surface before Brownian coagulation produced a significant reduction in agglomerate strength. About 40% of all bonds could not be broken at all in the untreated particles. After removing the highly volatile and the majority of the low volatile organic fraction of the adsorbate in the denuder, the remaining percentage of unbreakable bonds decreased to about 25%. The fragment size at maximum breakage was roughly equivalent to the size of the primary agglomerates coming from the engine. This suggests that those bonds formed after the immediate exhaust zone can be broken, and that the degree of breakage is sensitive to the amount of adsorbate.

**Figure 5:**
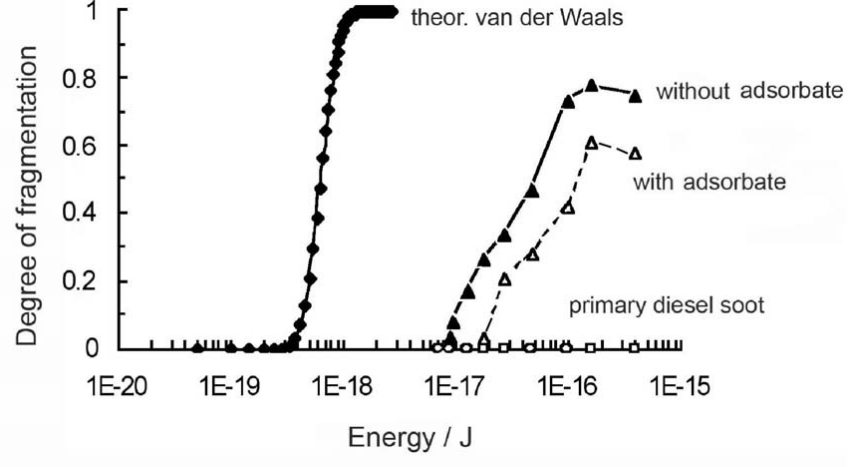
***Degrees of fragmentation as a function of the kinetic impact energy ***for aged agglomerate particles of diesel exhaust aerosol, measured with and without thermal denuder (TD). Also shown for information is the data for the primary diesel soot aerosol, which cannot be fragmented at all. The theoretical van-der-Waals bond energy distribution was calculated for a typical diesel soot agglomerate. (Note that the kinetic energy is based on the mass mean primary particle diameter. The diagram thus represents a bond energy distribution of primary particles.)

In Fig. 5, the experimentally measured fragmentation curves for aged diesel soot with and without TD are plotted against the respective impaction energy, calculated from the impact velocity on the basis of mass mean primary particle size (22 nm; compare Fig. 2) with an effective density of 1.5 g/cm^3 ^[19]. The 50% value in the diagram thus represents the mean energy required to break up a particle. (Presumably this mean fragmentation energy is somewhat higher than the mean bond energy of the agglomerate. However we still do not know how much additional energy goes into deformation and other secondary mechanisms.) – The 50% fragmentation energies taken from Fig. 5 are 5.2*10^-17 ^J with TD and 1.2*10^-16 ^J without TD. "Dried" diesel soot has thus roughly half the bond strength of diesel soot with adsorbates.

Recently, measurements of the bond strengths of unsintered and partially sintered silica nanoparticle agglomerates were made with the same experimental technique [Seipenbusch, M, Rothenbacher S, Kirchhoff M, Schmid HJ, Weber AP and Kasper G: Interparticle forces in silica nanoparticle agglomerates (submitted)]. The experiments were conducted by first forming van der Waals agglomerates by Brownian coagulation, and the heating these briefly to defined temperatures. Interestingly, they found very similar bond energies on the order 0.5 to 1*10^-16 ^for the silica particles just before the onset of sintering.

One can also make comparisons with calculated bond energies, keeping in mind however, that the model does not account for energy dissipated by rearrangement and deformation of an agglomerate during impact. This latter energy is significant [20] but very difficult to calculate with simple techniques. The theoretical distribution of bond energies in a van der Waals agglomerate was estimated according to a procedure described earlier [6] assuming a Hamaker constant of 23.8*10^-20 ^J [21], an average coordination number of 2.8 and the measured size distribution of the primary particles as shown in Fig. 2.

The calculated van der Waals bond energy distribution in a typical soot agglomerate with 90 primary particles as shown in Fig. 5 has a mean of 5.5*10^-19 ^J. The measured fragmentation energies are thus larger by factors of about 95 and 220 respectively. While this may appear very large, our results are nevertheless comparable with the findings of Rong et al. [4], who measured the forces required to break nanoagglomerates by AFM and also found ratios on the order of 100 to der pure van der Waals force.

A final point of interest is the fact that the fragmentation curve with adsorbates retains the shape of the curve without adsorbates. The shift to higher energies with concurrent loss of maximum degree of fragmentation can therefore be interpreted as resulting from increased interparticle energies, rather than an increase in coordination number (i.e. more compact agglomerates). An increased coordination number would also incur a shift of the curve to higher energies, but the same degree of fragmentation would eventually be reached. Indirectly, this would also suggest that the thermodesorption process did not substantially change the structure of the soot agglomerate.

## Summary and conclusion

The mechanical stability of airborne diesel soot particles was studied by determining fragmentation probabilities of individual agglomerates vs. impact velocity in a device specially designed to fragment particles directly from the aerosol phase.

The primary diesel soot was produced from an idling Volkswagen engine. Furthermore, the primary aerosol could be aged, i.e. subject to further agglomeration by Brownian coagulation. This could be done optionally with soot aerosol subject to a thermo-denuder to remove adsorbates at 280°C prior to aging.

It was found that the primary soot agglomerates emitted directly from the exhaust did not break-up at all, with the exception of a few very minor fragments, even at the highest impact velocities (300 m/s) achievable in the measurement device.

Soot agglomerates aged by secondary agglomeration could be broken down by impact fragmentation to roughly the size of the primary soot agglomerates. The presence of adsorbates had a significant influence on the energy required for fragmentation at this stage. The entire fragmentation curve was shifted to lower energies by a factor of roughly 2 for the "dried" soot. A maximum of 75% of bonds could be broken up in dried soot, versus only 60% without thermodesorption. (The organic carbon mass fraction of the primary soot ranged from about 10 to 25%.)

The average fragementation energies for dry and "wet" soot were determined to 0.55 and 1.2*19^-16 ^J. This compares well with measurements for unsintered inorganic nanoparticle agglomerates (Seipenbusch, M, Rothenbacher S, Kirchhoff M, Schmid HJ, Weber AP and Kasper G: Interparticle forces in silica nanoparticle agglomerates (submitted)).

The ratio of the measured average fragmentation energy of an agglomerate to the bond energy calculated on the basis of pure van der Waals interparticle forces was on the order of 10^2^. This also agrees with observations by Rong et al. (2006) [4] made with AFM.

Although a rigorous link between the fragmentability of agglomerate particles in the airborne state and their behavior in liquid suspension (such as cellular or other body fluids) remains to be established, these findings suggest that the smallest units to which diesel particles can be broken down are probably on the order of the size of primary emitted soot or larger.

## Competing interests

The authors declare that they have no competing interests.

## Authors' contributions

SR and AM performed the experiments; SR evaluated the data; GK wrote the paper.
